# Pattern and outcome of perforated peptic ulcer disease patient in four teaching hospitals in Addis Ababa, Ethiopia: a prospective cohort multicenter study

**DOI:** 10.1186/s12893-020-00796-7

**Published:** 2020-06-15

**Authors:** Jatani Arero Bupicha, Hailu Wondimu Gebresellassie, Abebe Alemayehu

**Affiliations:** grid.7123.7 0000 0001 1250 5688 CHS, SOM, AAU, Addis Ababa, Ethiopia

**Keywords:** Perforation, Peptic ulcer disease, Morbidity, Mortality, Pattern

## Abstract

**Background:**

Perforated peptic ulcer disease is a surgical emergency with a high morbidity and mortality. The socio-demographic characteristic and the factors associated with morbidity and mortality seems to differ between the developed and developing world. This is the first a prospective cohort study in Ethiopia designed to analyze pattern and outcome of patients with perforated peptic ulcer disease in four teaching hospitals affiliated with SOM, CHS of Addis Ababa University.

**Method:**

This is a prospective cohort study of patients operated for perforated peptic ulcer disease from June 1, 2018 to May 31, 2019 in four teaching hospital affiliated to department of surgery of SOM, CHS of Addis Ababa university.

**Result:**

A total of 97 patients were operated in a year. 86.6% were males with a male to female ratio of 6.5:1. The age group 21–30 were most affected constituting 42.3% of all patients. Mean age is 31.9, Median of 27, age ranges from 16 to 76. Alcohol use (45.4%) and previous history of ulcer disease (75.3%) were the most prevalent risk factors.33% were smokers. Abdominal was present in all and most presented within 48 h (79.4%). 85.6% had pneumo = peritoneum in an x-ray at presentation. Size of the perforation is 10 mm or less in 81.3%. 91(93.8%) had anterior first part duodenum perforation. Repair with pedicled omental patch was done in 65 (67.1%) patients. Age, duration of presentation, hypotension at presentation, size of perforation, degree of peritoneal contamination were found to be the significant factors for morbidity and mortality. Major morbidities were observed in 16 (16.5%) and mortality occurred in 3 (3.1%) patients.

**Conclusion:**

Perforation of peptic ulcer disease here occurs in the young. Age, duration of presentation, hypotension at presentation, size of perforation, degree of peritoneal contamination were found to be the significant factors for morbidity and mortality. Morbidity and mortality rate of 16.5 and 3.1% observed here are quite acceptable.

## Background

The natural history of peptic ulcer disease ranges from spontaneous resolution without intervention to development of life threatening complication such as bleeding and perforations. Perforated peptic ulcer is the 2nd most common ulcer related emergency following bleeding [1].

Perforated peptic ulcer is a surgical emergency and is associated with short-term mortality in up to 30% of patients and morbidity in up to 50% [1]. Worldwide variations in demography, socioeconomic status, Helicobacter pylori prevalence, and prescription drugs make investigation into risk factors for perforated peptic ulcer difficult [2].

Peptic ulcer related morbidity and mortality decreased in the western world since the mid-twentieth century especially in the young but ulcer mortality in senior citizens has, none the less, remained essentially unchanged or even increased [3].

The situation is different in the developing part of the world like Ethiopia where perforated PUD remains to be one of the top causes of acute abdomen and emergency surgery. Recent publication from Ethiopia put perforated peptic ulcer disease to be 3rd most common cause of acute abdomen following appendicitis and intestinal obstruction. More over our patients are younger, males are affected much more than females and the vast majority of perforations are duodenal [4, 5].

Most of the risk factors associated with perforation such as alcohol, smoking are well known but some may be related to local habits such as use of khat (*Catha edulis*), a stimulant leaf widely used in east Africa and Arabian peninsula and known for many gastrointestinal adverse effects [6].

Various techniques of closure (and their modifications) of the perforation were described such as simple closure, closure with vascularized omental pedicle (Cellon-Jones) and free omental plug (Graham’s). Putting a sub-hepatic drain after closure is being practiced in some centers like ours although its value is not substantiated [7].

The situation of the patient at presentation, delay at presentation and surgical intervention are now well known to be related to outcome of the patient [8].

## Methods

### Ethics approval and consent to participate

Ethical approval were gained from research and publication committee of department and approved by school of medicine in May 2018 reference number Dept. of surgery/SOM/61. Patients were counselled and written agreement obtained.

### Patient selection

All consecutive patients operated for perforated peptic ulcer disease in the four teaching hospitals (Minilik hospital, Yekatit-12 hospital, Zewditu Memorial Hospital, Tikur Anbessa specialized hospital) affiliated to department of surgery of school of medicine from June 1, 2018 to May 31, 2019 are included in the study.

### Patient management

All patients were initially resuscitated with crystalloids, nasogastric tube inserted, urinary bladder catheterized and metronidazole and 3rd generation cephalosporin often ceftriaxone started. Informed consent for emergency surgery obtained. Laparotomy done and the intra-abdominal situation assessed. The intra-peritoneal sucked out and a thorough lavage with warm saline done. Then the perforation were dealt with either free omental patch or a vascularized pedicled omental patch depending on the preference of operating surgeon.

### Statistical analysis

Patient demography, possible risk factors, status at presentation, intra-operative findings, post-operative complications and final outcome were collected and filled in to a pre-prepared format. Patients were followed from admission to discharge or death. The data were collected by surgical residents and interns and checked by the authors.

Statistical analysis was performed using IBM-SPSS-version-25 statistical package. The mean standard deviation (SD), median and ranges were calculated for continuous variables whereas proportions and frequency tables were used to summarize categorical variables. Chi-square test was used to test for the significance of association between the independent (predictor) and dependent (outcome) variables in the categorical variables. The level of significance was considered as P < 0.05.

## Results

### Patients’ background

During the study period 97 patients were operated. 84 (86.6%) were males and 13 (13.4%) were females with a male to female ratio of 6.5 to 1. Patients’ age ranged from 16 to 76, Mean age of 31.9, Median of 27. Only 15 (15.5%) came outside the city. 77.3% were orthodox Christians and 16.5% were Muslims. Regular alcohol use were reported by 44 (45.4%) and smoking in the third (33%) of all patients. Use of the local addictive stimulant khat (*Catha edulis*) were found in 17 (17.5%) of patients. Although 73 (75.3%) patients gave history of PUD the Helicobacter Pylori status of 90 (92.8%) were known at the time of presentation. Perforation occurred while fasting 13(13.4%) patients (Table 1).

**Table 1:** Socio-demographic data of patients with perforated Peptic ulcer disease in four teaching hospital in Addis Ababa, Ethiopia

Category		Number	Percentage
GENDER	Male	84	86.6
	Female	13	13.4
	Male: Female ratio 6.5: 1		
Age	Mean age of 31.9,Median of 27,Age range from 16 to 76		
	11–20	17	17.5
	21–30	41	42.3
	31–40	23	23.7
	41–50	4	4.1
	51–60	6	6.2
	61–70	4	4.1
	71–80	2	2.1
Address	Addis Ababa	82	84.5
	Outside Addis Ababa	15	15.5
Religion	Orthodox Christian	75	77.3
	Muslims	16	16.5
	Other Christians	6	6.2
Alcohol use	Yes	44	45.4
	No	53	54.6
Khat use (*Catha edulis*)	Yes	17	17.5
	No	80	82.5
Smoking	yes	32	33
	No	65	67
Co-morbidity	present	3	3.1
	Absent	94	96.9
NSAID use	Present	2	2.1
	absent	95	97.9
Fasting	present	13	13.4
season	summer	18	18.6
	autumn	19	19.6
	winter	26	26.7
	spring	34	35.1
History of PUD *H. pylori* infection	Present	73	75.3
	absent	24	24.7
	positive	3	3.1
	Negative	4	4.1
	unknown	90	92.8

Forty four (45.4%) presented within 24 h, 33 patients presented between 24 and 48 h after onset of abdominal pain. Abdominal pain were the presenting complaint in all patients and physical examination revealed generalized peritonitis in 85 (87.6%) patients. 17 (17.5%) had hypotension and raised WBC count were observed in 45 (46.4%). X-ray evidence of free peritoneal air were found in 83 of the 97 (85.6%) patients (Table 2).

**Table 2:** Clinical and operative findings of patients with perforated Peptic ulcer disease in four teaching hospital in Addis Ababa, Ethiopia

Clinical presentation		number	%
Presenting complaint	Abdominal pain	97	100
	vomiting	82	84.5
	Abdominal distension	78	80.4
Duration of presentation	< 24 h	44	45.4
	24–48 h	33	34
	48–72 h	10	10.3
	> 72 h	10	10.3
Physical finding	Generalized peritonitis	85	87.6
	Localized peritonitis	12	12.4
Hypotension at presentation	present	17	17.5
	absent	80	82.5
WBC count at presentation	leukocytosis	45	46.4
	normal	52	53.6
Chest x ray finding	positive	83	85.6
	negative	11	11.3
	Not done	3	3.1
Size of perforation	< 10 mm in diameter	79	81.3
	10–20 in diameter	12	12.4
	> 20 mm diameter	2	2.1
Location of perforation	duodenal	91	93.8
	Pre-pyloric/gastric	2	2.1
	Sealed ant duodenal	4	4.1
Degree of peritoneal contamination	< 1 l	72	74.2
	1–2 l	21	21.6
	> 3 l	4	4.1

### Intraoperative finding and procedure done

The location of the perforation were in the first part of duodenum anteriorly in 91 (93.8%) and the size of the perforation was less than 10 mm in diameter in 79 (81.3%). Less than a liter of peritoneal GI content were found in 72 (74.2%) patients.

Repair with vascularized omental patch were performed in 65 (67.1%) and free omental patch in 28 (28.8%) patients (Table 2 and Table 3).

**Table 3:** Method of repair perforated Peptic ulcer disease in four teaching hospital in Addis Ababa, Ethiopia

Repair method	Number	%
Simple repair	0	0
Repair with omental reinforcement	65	67.1
Graham’s omental patch	28	28.8
Sealed no patch needed	4	4.1
Sub-hepatic drain inserted	89	91.8

### Postoperative outcome

Major complications such as surgical site infection, pneumonia, post-operative collection and patch failure were observed in 16 (16.5%) of the patients. 94 (96.6%) were discharged improved and 3 (3.1%) died. The majority (53 patients or 54.6%) were discharged within 7 days (Table 4).

**Table 4:** Morbidity mortality, hospital stay duration patients with perforated Peptic ulcer disease in four teaching hospital in Addis Ababa, Ethiopia

Variables		Number	%
Hospital stay	< 7 days	53	54.6
	7–14 days	33	34.0
	> 14 days	11	11.3
Morbidity	Patch failure	5	5.2
	SSI	5	5.2
	pneumonia	5	5.2
	Post-operative collection	1	1.1
	Re-laparotomy (for patch failure & recollection)	5	5.1
	Total	16	16.5
outcome	Discharged improved	94	96.6
	Died	3	3.1

Analysis in terms of morbidity and mortality of the possible risk factors showed higher age (> 40 years), longer duration at presentation (> 48 h), hypotension at presentation, higher degree of peritoneal contamination were significant risk factors (P < 0.05) (Table 5).

**Table 5:** Factors influencing morbidity and mortality on bivariate logistic regression of patients with perforated Peptic ulcer disease in four teaching hospital in Addis Ababa, Ethiopia

Variable		Complications N (%) *N* = 16	No Complications N (%) *N* = 68	*P*-value	Death N (%) *N* = 3	Discharged improved N (%) *N* = 94	*P*-value
Sex	Male	16 (19.04)	68 (80.1)		3(3.6)	81(96.4)	
	Female	0 (0)	13(100)	0.136	0	13	0.787
Age	11–20	3 (17.6)	14 (82.4)		0 (0)	17(100)	
	21–30	4(9.8)	37 (90.2)		0(0)	41(100)	
	31–40	4 (17.4)	19 (82.6)		2 (8.7)	21(91.3)	
	41–50	1 (25)	3 (75)		0(0)	4 (100)	
	51–60	1 (16.7)	5 (83.3)		0 (0)	6(100)	
	61–70	2 (50)	2 (50)		1(25)	3(75)	
	71–80	1 (50)	1 (50)	0.08	0(0)	2 (100)	0.003
Address	AA	12 (14.6)	70(85.4)		3 (3.6)	79(96.4)	
	Outside	4 (26.7)	11(73.3)	0.391	0 (0)	15(100)	0.475
Religion	Orthodox	11(14.9)	63(85.1)		2 (2.7)	73 (98.3)	
	Muslim	4(25)	12(75)		1 (6.3)	15 (93.7)	
	Others Christians	0(0)	6(100)	0.310	0 (0)	6 (100)	0.746
History of PUD	History of PUD	13 (17.8)	60 (82.2)		3 (4.1)	70 (95.90	
	No history of PUD	3 (12.50	21(87.50		0 (0)	24(100)	0.337
Smoking	Yes	5 (15.6)	27(84.40)		0 (0)	32(100)	
	No	11(16.9)	54 (84.10	0.922	3 (4.6)	62 (95.4)	0.240
Alcohol	Yes	4 (16.4)	38(83.6)		0 (0)	44 (100)	
	No	10 (18.70)	43 (81.3)	0.212			0.126
Khat use/Catha edulis	Yes	2 (12.5)	14 (87.5)		1(5.9)	16 (94.1)	
	NO	15 (18.5)	66 (81.5)	0.141	2 (2.5)	78 (97.5)	0.323
Co-morbidity	Yes	0 (0)	3 (100)		1(33.3)	2 (66.70	
		16 (17)	78(83)				0.00
NSAID use	yes	1 (50)	1(50)		0 (0)	2 (100)	
	No	15 (15.8)	80 (84.2)	0.076	3 (3.2)	92(96.8)	0.809
Hx of PUD	Yes	13(17.8)	60 (82.2)		3 (4.1)	70 (95.9)	
	No	3 (12.5)	21(87.5)	0.139	0	0	
H.Pylri infection	Present	1 (33.3)	2 (66.7)	0.647	1 (33.3)	2 (66.70	0.02
Duration at presentation	< 24 h.	4 (9)	40 (91)		0(0)	44 (100)	
	24–48 h.	5 (15.1)	28 (84.9)		1 (3)	32 (97)	
	48–72 h.	3 (27.3)	7(72.7)		0(0)	10 (100)	
	> 72 h.	4 (40)	6 (60)	0.004	2 (20)	8 (80)	0.003
WBC count	Raised	7 (15.6)	38 (84.4)		0 (0)	45 (100)	
	Normal	9 (17.3)	43 (82.7)	0.610	3 (5.8)	49 (94.2)	
CXR finding	Free air	14 (16.7)	69 (83.3)		2 (2.4)	81(97.6)	
	Not detected	1(9.1)	10 (90.9)		0 (0)	11(100)	
	Not done	1 (33.3)	2 (66.7)		1 (33.3)	2 (66.7)	
Hypotension	Yes	9 (52.9)	8 (47.1)	0.000	3 (17.6)	14 (82.4)	0.001
Perforation size	< 10 mm	7 (9.2)	69 (90.8)		0 (0)	79 (100)	
	10-20 mm	5 (41.7)	7 (59.3)		1 (8.3)	11(91.7)	
	> 20 mm	1 (50)	1 (50)		2 (100)	0 (0)	0.000
	Sealed	0 (0)	4 (100)	0,046	0 (0)	1(100)	
contamination	< 1 Lts	7 (9.7)	65 (90.3)		1 (1.4)	71 (98.6)	
	1–3 Lts	7 (33.3)	14 (66.7)		1 (4.8)	20 (95.2)	
	> 3 Lts	1 (33.3)	2 (66.7)	0.000	1 (25)	3 (75)	0.006
Procedure done	Simple closure	0 (0)	0 (0)		0 (0)	0 (0)	
	Pedicled omental	11(16.9)	54 (83.1)		3 (4.6)	62(95.4)	
	Free omental	5 (18.5)	23 (81.5)	0.399	0 (0)	27 (100)	
Drainage left		15 (16.8)	74 (83.2)		3 (3.4)	86 (96.6)	

## Discussion

This study showed that male to female ratio of patients with perforated peptic ulcer disease is 6.5 to 1.0. This is similar the two studies done in hospitals here in Addis Ababa by Assefa Z and Ersemo T where the male to female ratio was reported to be 6.6: 1and 5.6:1.0 respectively. A study by Phillipo L Chalya et al. from Tanzania showed the male to female ratio of only 1.3 to 1 [9, 10]. This difference in incidence do not seem to be the case in developed countries as a study by Thorsen K et al. on epidemiology of perforated peptic ulcer disease in Norway showed that females are affected more than males (that 89 of 172 of their patients were females) [11].

The mean age of patients in this study is just 31.9 and 42.3% of our patients were aged between 21 and 30 suggesting that our patients here in Ethiopia are much younger than most other reports. Two studies from Nigeria (one on Five-Year Review of Perforated Peptic Ulcer Disease in Irrua, Nigeria by A. E. Dongo et al. and another on Audit of Perforated Peptic Ulcer Disease in a Tropical Teaching Hospital) showed the pick age of their patients to be on 5th decade [12, 13] and patients with PUD perforation in developed countries are even more older as seen by the study in Norway by Thorsen K et al. (68% of their patients were aged 60 years or older).

Similar to a previous study by Abebe B in Minilik II hospital, most of our patients (84.5%) came from the capital city Addis Ababa [14].

A regular use of alcohol and smoking were found in 45.4 and 33% of our patients respectively. A study in a tertiary hospital in Tanzania 85.7% use alcohol and 64.3% were smokers. A study from eastern India by Nishith M Paul Ekka and Shital Malua also reported 65.73% were known smokers while 42.86% patients were admittedly alcoholics [15, 16].

Consumption of the stimulant leaf Khat (Catha edulis) which is wide spread in eastern Africa and Arabian Peninsula were found in nearly one in five of our patients (17.5%) with PUD perforation. This is much lower than the finding in a study on pattern and seasonal variation of PUD perforation in this city by Abebe B and his colleagues where 50.5% were found to be a regular consumer. Another study from Zewditu Memorial hospital by Assefa Z and G/eyesus A. showed 75% of their patients’ with PUD perforation use Khat. The use of this substance is associated with a number of gastrointestinal problems [9, 14].

75.3% of our patients gave history of PUD and this is similar to reports from Tanzania where 69% had PUD history but the study in Irrua, Nigeria 59.6% had no history of peptic ulcer disease [17]

Unlike the studies in developed countries like Norway comorbidities and use of NSAID were found in only in 3.1 and 2.1% respectively in this study but a study in Ghana by J. C. B. Dakubo and his colleagues revealed a regular ingestion of NSAIDS in 47.7% patients and 18.2% had associated co-morbid conditions [18].

Among the seasons of the year perforation occurred more in the spring (March 1 to May 31) which happens to be the fasting months for both Orthodox Christian and Muslims in Ethiopia but only 13.4% were fasting when the perforation occurred. This has been reported to be the case in some studies [19].

Abdominal pain were the presenting complaint in all of our patients followed by vomiting and abdominal distension similar to finding Nigerian study by A. E. Dongo et al. Nearly half (45.4%) our patients came within 24 h of onset this is similar to the study in in Accra, Ghana but a study on presentation and management of Perforated Peptic Ulcer Disease in a Tertiary Centre In South Nigeria by Dodiyi-Manuel A et al. only 11.1% patients presented within 24 h of the onset of symptoms [17, 20].

Generalized peritonitis were present on 85 of 97 patients (87.6%) in this study and this is similar to the study in eastern India where 97.8% of their patients had abdominal rigidity or guarding. The study from Tanzania also showed that abdominal tenderness and classical signs of peritonitis were demonstrable in 88.1 and 66.7% patients respectively [18].

In this study hypotension at presentation were observed in only 17 of 97(17.5%) patients but the Tanzanian study reported shock occurred in 33.3% of their patients. Leukocytosis were found in 45/97 our patients (46.4%) but 91.2% had leukocytosis in the Indian study [16].

Air under diaphragm was found in 83 of the 97(85.6%) patients in this study similar a clinical study of peptic ulcer perforation in eastern India where 82.41% patients showed radiological sign of gas under diaphragm [13, 16].

The location of the perforation is first part of duodenum in 93.8% of our patients. This is similar to the study in Northwestern Tanzania where most perforations were located on the duodenum {92.9%), whereas in the remaining 7.1% patients had their ulcers located on the stomach. Reports from Norway, The Netherlands and Iceland showed gastric ulcer perforation predominate over duodenal perforations in that part world [21].

The size of the perforation is < 10 mm in diameter in 81.3% of our patients but this was not the finding in a study on Clinical profile and outcome of surgical treatment of perforated peptic ulcers in Northwestern Tanzania where thirteen (15.5%) of the perforations were of minimal size (≤5 mm) and sixty-four (76.2%) were massive (> 10 mm). Another study from in Irrua, Nigeria showed 49% of perforations had a diameter of < 10 mm [22].

Repair with pedicle omental patch was done in 67.1%, free Grahams omental patch in 28.8% and 4 (4.1%) were found to have a sealed perforation in this study. Graham’s Omentopexy was procedure of choice in Nigerian study accounting for 69.2%. Ibadan Nigeria Simple closure was performed in 25%, pedicled omental plug done in 60% and primary closure with an onlay omentum in 15% of the patients [18, 20].

In this study significant morbidities (such as patch failure, pneumonia, SSI) occurred in 16 of 97 patients (16.5%) and this is acceptable and lower than most reports from Africa. We have three postoperative deaths making mortality rate 3.1% similar to SeungJin et al. where an overall 30-day mortality rate to be 3.17%.(The statistically significant factors associated with increased morbidity and mortality in this study with a P-value less than 0.05 were older age, hypotension at presentation, delay at presentation and perforation greater than 10 mm in diameter. In a paper that summarize the evidence for perforated peptic ulcer management and identify directions for future clinical research in the Lancet all of the above were described to significant factors for postoperative morbidity and mortality in patients with PUD perforation [8, 23].

## Conclusion

Perforation of peptic ulcer disease here occurs in the very young. Age, duration of presentation, hypotension at presentation, size of perforation, degree of peritoneal contamination were found to be the significant factors for morbidity and mortality. Morbidity and mortality rate of 16.5 and 3.1% observed here quite acceptable.

## Data Availability

The original data collected and compiled SPSS file is available with corresponding author.

## Abbreviations

SOM: School of medicine
CHS: College of health sciences
AAU: Addis Ababa university
GI: Gastrointestinal
PUD: Peptic ulcer disease
AA: Addis Ababa
